# Oxygen depletion in coastal seas and the effective spawning stock biomass of an exploited fish species

**DOI:** 10.1098/rsos.150338

**Published:** 2016-01-13

**Authors:** H.-H. Hinrichsen, B. von Dewitz, J. Dierking, H. Haslob, A. Makarchouk, C. Petereit, R. Voss

**Affiliations:** 1 GEOMAR – Helmholtz Centre for Ocean Research, Düsternbrooker Weg 20, 24105 Kiel, Germany; 2 Thünen-Institute of Sea Fisheries, Palmaille 9, 22767 Hamburg, Germany; 3 Fishery Resources Research Department, Institute of Food Safety, Animal Health and Environment ‘BIOR’, Daugavgrivas 8, Riga 1048, Latvia; 4 Sustainable Fishery, Department of Economy, University of Kiel, Wilhelm-Seelig-Platz 1, 24118 Kiel, Germany

**Keywords:** neutral egg buoyancy, Bornholm Basin, habitat suitability, oxygen-related cod egg survival, recruitment

## Abstract

Environmental conditions may have previously underappreciated effects on the reproductive processes of commercially exploited fish populations, for example eastern Baltic cod, that are living at the physiological limits of their distribution. In the Baltic Sea, salinity affects neutral egg buoyancy, which is positively correlated with egg survival, as only water layers away from the oxygen consumption-dominated sea bottom contain sufficient oxygen. Egg buoyancy is positively correlated to female spawner age/size. From observations in the Baltic Sea, a field-based relationship between egg diameter and buoyancy (floating depth) could be established. Hence, based on the age structure of the spawning stock, we quantify the number of effective spawners, which are able to reproduce under ambient hydrographic conditions. For the time period 1993–2010, our results revealed large variations in the horizontal extent of spawning habitat (1000–20 000 km^2^) and oxygen-dependent egg survival (10–80%). The novel concept of an effective spawning stock biomass takes into account offspring that survive depending on the spawning stock age/size structure, if reproductive success is related to egg buoyancy and the extent of hypoxic areas. Effective spawning stock biomass reflected the role of environmental conditions for Baltic cod recruitment better than the spawning stock biomass alone, highlighting the importance of including environmental information in ecosystem-based management approaches.

## 1. Introduction

Large-scale expansion of oxygen minimum zones (OMZs) in marine systems and large lakes was observed worldwide over the past decades, and it is a challenging task to predict future impacts on fish stocks and their ecosystems [1,2]. Oxygen reduction could lead to widespread mortality [2] or avoidance of affected areas [3–5]. Thus, OMZs may have detrimental effects on the reproductive processes of mass-spawning, commercially exploited fish populations. OMZs will have major ecological and economic implications in the future [6] and are expected to further increase as an aspect of global climate change, in particular in northern latitudes including the Baltic Sea [2,7,8].

A typical example for a semi-pelagic fish stock in coastal habitats suffering from oxygen reduction is the eastern Baltic cod (*Gadus morhua*) population. This stock is historically the third largest Atlantic cod stock [9] with a long-term mean spawning stock biomass between 1966 and 2012 of about 240 000 tonnes [10]. The eastern Baltic cod stock spawns in the major deep basins (Bornholm Basin, Gdansk Deep and Gotland Basin), which are characterized by a permanent haline stratification [11,12]. The principal mechanism causing replenishment of oxygen in these deep Baltic Sea basins is the ephemeral nature of ‘Baltic inflows’ [13] of highly saline oxygen-rich water masses from the North Sea. Owing to the effect of human-induced eutrophication and climate-induced less frequent inflow events, the oxygen conditions in recent decades became less favourable, and hypoxic areas have expanded [14]. A corresponding environmentally dependent low recruitment success combined with high fishing pressure resulted in a reduction of the spawning stock biomass of eastern Baltic cod from almost 650 000 to 87 000 tonnes from 1983 to 1992.

Many marine fish species including cod in the Baltic Sea are living at the physiological limits of their distribution, owing to the strong gradient from fully marine salinities in the western Baltic to brackish water salinities in the northeast. For example, salinity is correlated with neutral egg buoyancy [15]. Neutral buoyancy for Baltic cod eggs is found at salinities which typically occur from below the halocline to the bottom water [16–18].

The interaction between egg buoyancy (i.e. floating depth) and oxygen concentration is important for the survival of eggs of different species in the Baltic Sea [16,19]. Specifically, owing to the hydrography of the Baltic Sea, the development of more buoyant eggs occurs in more favourable oxygen conditions. Egg buoyancy is positively correlated to egg diameter, which in turn is influenced by spawner characteristics. Kjesbu *et al.* [20,21] showed a highly significant positive correlation between egg diameter and female size for Atlantic cod. For Baltic cod several experimental studies confirmed that large, older females, on average, produced larger eggs with neutral buoyancy at lower salinity (water density) compared with smaller eggs spawned by smaller, younger females [16,22]. Although egg size also varies within a batch, and between batches produced throughout the spawning season, larger more buoyant eggs spawned by larger (older) females [23] are hence expected to have on average higher egg survival probability.

Here, the presented approach follows the need to inform Integrated Assessments and Ecosystem-based Management [24]. Within this context, analyses on the state and development of the various Baltic ecosystems have been conducted [25–28]. The ICES Working Group on Integrated Assessments of the Baltic (WGIAB) has successfully performed integrated trend and status assessments which have been used for developing a set of ecosystem indicators that can support the assessment of the stock status of individual species [29]. Individual indicators were evaluated for showing support for or against the assessment model-derived stock status and trends.

Here, we provide the first comprehensive, field-based evidence for the link between cod egg size and floating depth. We then quantify and visualize the stock-structure effect on spawning area extension, the ambient oxygen concentration experienced by eggs as well as egg mortality for contrasting environmental conditions (i.e. post-inflow versus stagnation). In a third step, we calculate timeseries of oxygen-related cod egg survival probability for the entire spawning periods from 1993 to 2010. Finally, we develop and test the concept of effective spawning stock biomass (eSSB) as an indicator of recruitment potential, which is integrating biomass, stock structure and environmental conditions, with the eastern Baltic cod stock as an example.

## 2. Material and methods

### 2.1 Field validation

**Figure 1.**
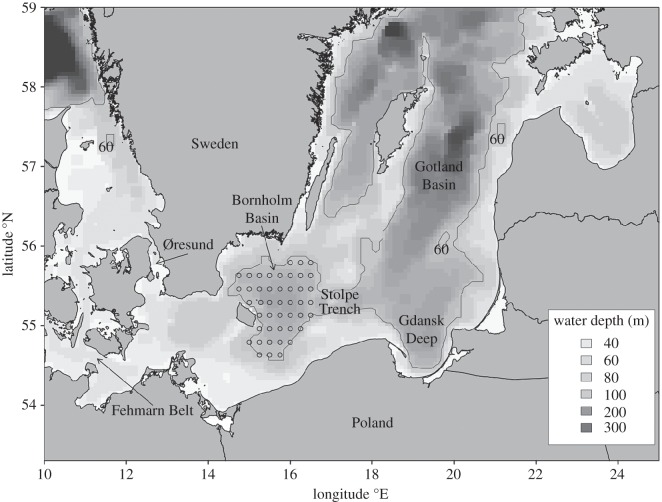
Map of the Baltic Sea with standard station grid in the Bornholm Basin (45 stations).

In order to confirm a laboratory-derived correlation between egg size and floating depth (i.e. water density) for variable hydrographic conditions in the field, vertically resolved sampling was conducted during seven RV *Alkor* cruises between 2002 and 2010. The vertical distribution of cod eggs was investigated with a towed multiple opening–closing net at selected stations with large numbers of cod eggs as derived from Bongo net samples (figure 1). The gear in use was a HYDROBIOS MAXI-type Multinet (Hydrobios, Holtenau, Germany) with a net mouth opening of 0.5 m^2^ equipped with net sets with 335 μm mesh size. At each station, depth profiles covering the whole water column from the surface down to near bottom were sampled in 5 m intervals. Samples were preserved in 4% buffered formaldehyde/seawater solution immediately after collection, and cod egg diameters (in 0.025 mm size classes) were subsequently measured for at least 100 eggs per sample. Weighted mean egg diameters per sample were then calculated and assigned to mean ambient water densities/egg buoyancies as derived from hydrographic measurements with a CTD probe (conductivity, temperature, depth) taken at each of the sampling stations. As a next step, from a total of 150 multinet samples, average egg diameters were correlated to water density (measured in classes of 0.5 kg m^−3^).

### 2.2 Quantification of the spawning environment and egg mortality

To calculate the spatial extent of the spawning environment, hydrographic data (temperature, salinity, oxygen concentration) were compiled from 54 cruises in the Bornholm Basin between 1993 and 2010. The surveyed area displayed by the station grid in figure 1 covers the area enclosed by the 60 m isobaths, which represents the historical peak egg and larval abundance area of Baltic cod in the Bornholm Basin [30]. The cruises consisted of a total of 45 standard stations, with a horizontal resolution of 8–10 nautical miles. In only a few years, not at all stations were hydrographic measurements available, mainly owing to bad weather conditions. However, a sufficient horizontal coverage of hydrographic properties for further analyses could be achieved for all years. Based on hydrographic parameters measured at the standard station grid, for each cruise, we calculated the three-dimensional extension of the spawning volume. As obtained from field observations as well as from laboratory experiments for eastern Baltic cod, a minimum salinity level of 11 psu is required for neutral egg buoyancy, whereas eggs at lower salinities sink to the bottom and die. At the same time, a minimum oxygen level of 2 ml l^−1^ and temperatures above 1.5°C are required for eggs to develop successfully [17,18,31].

We displayed the horizontal distributions of the spawning conditions for different levels of egg buoyancy, which is associated with female spawner size. The distributions were estimated with an objective analysis [32]. This method is designed for datasets containing relatively low numbers of observations and is able to interpolate across stations where no data could be assigned. It is based on a standard statistical approach, the Gauss–Markov theorem, which gives an expression for the linear least-square error estimate of the variables. The method creates horizontal fields representing the spawning conditions by interpolating the associated observed environmental parameters onto a regular grid (see electronic supplementary material, appendix S1 and equation S1). The method can make use of statistics with respect to measurement errors and small-scale noise inferred from the observed data. Thus, at single locations, an estimate can be given that depends linearly on the total number of measurements, i.e. a weighted sum of all observations. Based on repeated observations at individual stations, it was obtained that the error owing to measurement errors and small-scale noise amounts to 15% of the total variance of the abundance fields and that these errors are normally distributed. As most of the surveys were designed to produce quasi-synoptic horizontal fields of the Baltic cod spawning conditions, a unit array configuration with a horizontal resolution of 5 km was chosen and superimposed on the standard station grid. Thereby, each of the grid points is representative of the analysed properties around it. However, only areas were considered where the expected root mean square of the interpolation was less than 50% of the standard deviations of the observed property fields. Generally, the objective analysis method provides a smoothed version of the original measurements, with a tendency to underestimate the observed values. This is due to the specific assumptions made regarding our treatment of measurement noise and small-scale signals unresolved by the mesoscale observation array. The error estimates depend mainly on the statistics of the field, the noise level and on the locations of the observations, but not on the measurements themselves.

Oxygen-dependent survival probability maps were constructed for those density levels where cod eggs in the Baltic Sea are typically neutrally buoyant.

Horizontal maps and timeseries data on the cod spawning habitat characteristics were assembled for different buoyancy levels (1009, 1011 and 1013 kg m^−3^, corresponding to *ca* 11, 13.5 and 16 psu at a temperature of around 5°C). These levels were chosen to represent the buoyancies of eggs of different female age categories: old (large), mid-age (medium size) and young (small) cod, and were based on the results described in [23].

### 2.3 Calculation of effective spawning stock biomass

We used the information on variable egg mortality depending on spawner age and hydrographic conditions to derive a new timeseries of eSSB. This new timeseries can be seen as a derivative of stock reproductive potential and mirrors the effect of variable age distribution in the spawning stock in combination with variable hydrographic conditions on recruitment; it is tested and used in a stock-recruitment model. We tested which stock-recruitment function yielded the best fit for the traditional SSB estimates and the full timeseries 1966–2012. The Ricker-type model fitted the data best, being, however, only slightly better than the Beverton–Holt or the smoothed hockey stick model. Cod cannibalism [33] as well as food competition [34] also suggests a density-dependent model. Therefore, we concentrated on the Ricker model. Because, in our analysis, we were not able to account for all factors contributing to variations on the female size/egg buoyancy structure (see above), we calculated simplified egg buoyancy levels (averages over all spawning batches) for each female age class based on experimental studies [23], using data for peak spawning time (table 1).

**Table 1.** Mean egg density (averaged over all spawning batches) for female spawners of different age [23].

age class	egg density
8	1010±1.11
7	1010.71±0.85
6	1010.95±0.84
5	1011.25±0.82
4	1011.61±0.81
3	1012.06±0.42
2	1012.71±0.40

For the timeseries 1951–2012, we then calculated oxygen-dependent egg mortality for each year and each female spawner age class in May. Ambient oxygen contents were derived from the ICES hydrographic database and were inserted into an egg survival function [35] based on experimental data (equation S2 in appendix S2 in the electronic supplementary material).

**Figure 2.**
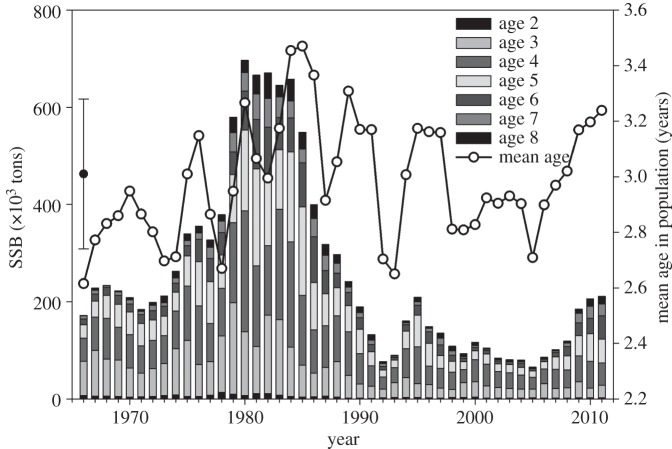
Baltic cod spawning stock size, its age composition and mean age 1966–2012. The symbol to the left indicates mean age and standard deviation over the investigated time period.

**Figure 3.**
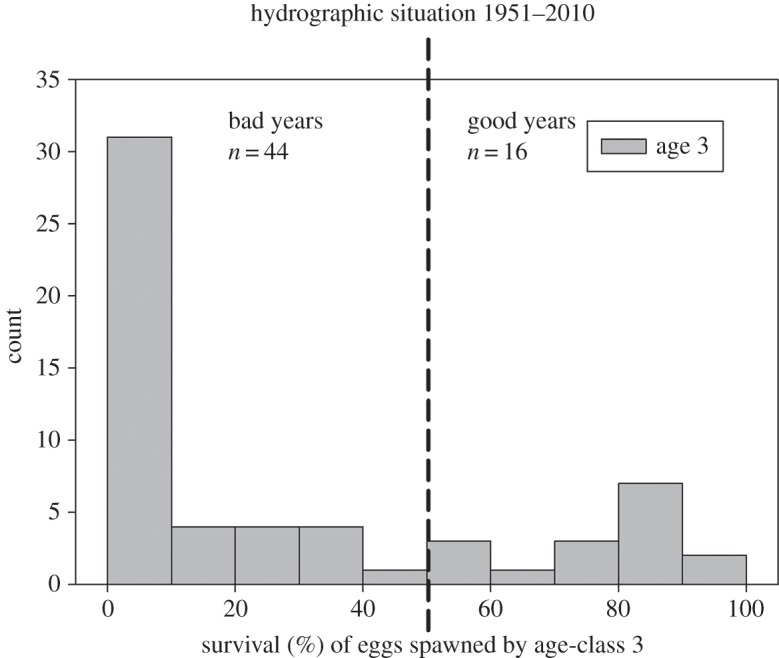
Survival of eggs spawned in May by age-class 3 in 1951–2010. Years with egg survival >50% (‘good’ years, *n* = 16) are 1952, 1954, 1959, 1960, 1961, 1964, 1969, 1972, 1976, 1979, 1980, 1986, 1993, 1994, 1998, 2003.

Hydrographic conditions in the depth range of floating cod eggs are mainly influenced by irregular inflow events of North Sea water. Inflowing waters replenish the oxygen content below the permanent halocline and hence are positive for cod egg survival. Owing to fast oxygen consumption caused by breakdown of organic material, good, post-inflow conditions usually last for only one year, i.e. the following year faces bad oxygen conditions again. Taking these hydrographic features into account, we divided the historic timeseries into ‘good’ versus ‘bad’ condition years (and therefore high and low cod egg survival). Mean age of the historic age distribution of the stock from 1966 to 2012 (figure 2) was 3.0 (±0.3) years, showing variations between 2.6 and 3.5 years. Therefore, we used egg survival at age 3 as distinctive feature, with egg survival more than 50% classifying a ‘good’ year and egg survival less than 50% classifying a ‘bad’ year. This resulted in a bimodal distribution, with 44 years being classified as ‘bad’ and 16 years with egg survival more than 50% (figure 3), classified as ‘good’ years.

For each of the two sets of years, we calculated mean age-specific egg survival (table 2).

**Table 2.** Mean egg survival for different female age classes under good (*n*=16 years) and bad environmental conditions (*n*=44 years) out of the timeseries 1951–2010.

age-class	survival (bad)	survival (good)
8	47.3	86.1
7	35.9	76.3
6	30.1	73.2
5	23.2	71
4	15.6	73
3	8.4	76
2	1.7	60.7

To calculate the eSSB timeseries, we used relative egg survival as weighting factor for potential recruitment contribution. The factor would be 1 at 100% egg survival. All age classes showed lower potential egg survival and received weighting factors between 0.861 (age-class 8+ under good conditions) and 0.017 (age-class 2 under bad conditions). For more detail, see appendix S3.

## 3. Results

**Figure 4.**
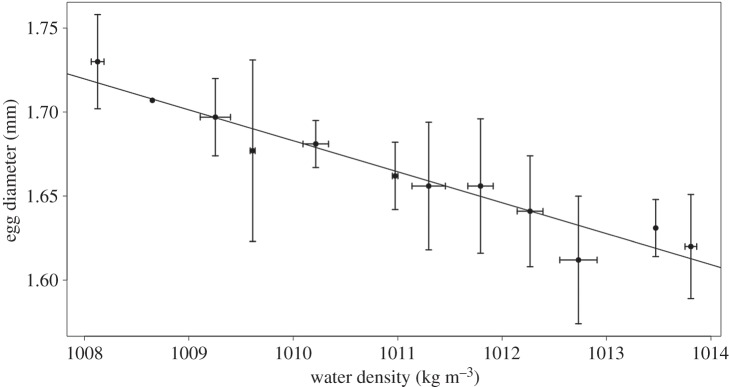
Mean egg diameter versus mean density of ambient seawater. Error bars represent standard deviation of mean values.

Cod egg mean diameter showed a strong negative correlation (*r*=−0.96) with the mean water density at which eggs were present in the field (figure 4), suggesting that egg diameter is directly associated with neutral buoyancy. This confirmed that laboratory-derived results are also applicable in the field, even under highly variable hydrographic conditions.

**Figure 5.**
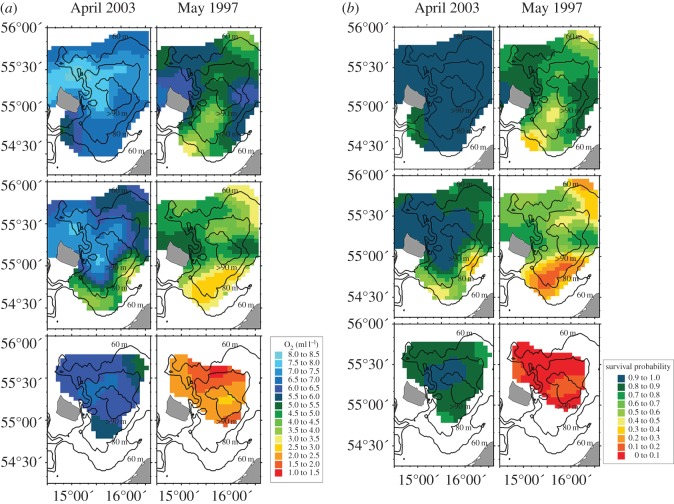
Horizontal maps of oxygen content in ml l^−1^ (*a*) and Baltic cod oxygen-related egg survival (*b*) for different water density (buoyancy)levels (1009, 1011, 1013 kg m^−3^) after a major Baltic inflow event in April 2003 and during a stagnation period in May 1997.

The horizontal maps showing the Baltic cod spawning habitat extension and corresponding oxygen-related cod egg survival revealed (i) strong differences in suitable habitat related to the different buoyancy levels and (ii) strong temporal fluctuations related to environmental variability (figure 5) between a major Baltic inflow situation in April 2003 and a stagnation period in May 1997. Specifically, after the major Baltic inflow in 2003, the areas related to the selected buoyancy levels (1009, 1011, 1013 kg m^−3^, corresponding to *ca* 11, 13.5 and 16 psu at a temperature of around 5°C) were widely extended over the whole Baltic cod spawning ground in the Bornholm Basin (17 872, 16 302, 8594 km^2^ on the respective buoyancy level) with overall almost optimal favourable spawning conditions in terms of cod egg survival probabilities (mean: 0.94, 0.83, 0.87). During the stagnation period the maps suggest a significant decrease of the extent of suitable spawning habitat (17 187, 15 617, 3826 km^2^) at the different egg buoyancy levels associated with on average much lower egg survival probabilities (0.73, 0.52, 0.09). Average habitat extension and egg survival probabilities for the different egg buoyancy levels are summarized in table 3.

**Table 3.** Monthly mean vertical habitat suitabilities and oxygen-related egg survival probabilities of eastern Baltic cod in the Bornholm Basin (1993–2010).

	neutral egg			
month	buoyancy (kg m^−3^)	area size (km^2^)	survival probability	*n*
April	1009	17 469±1299	0.85±0.10	18
May	1009	17 253±1249	0.74±0.13	18
July	1009	17 313±1517	0.63±0.16	18
April	1011	14 472±1807	0.53±0.21	18
May	1011	13 131±3217	0.42±0.21	18
July	1011	11 779±4652	0.32±0.22	17
April	1013	6321±5039	0.61±0.31	10
May	1013	4368±5324	0.49±0.24	10
July	1013	8886±3219	0.43±0.15	4

**Figure 6.**
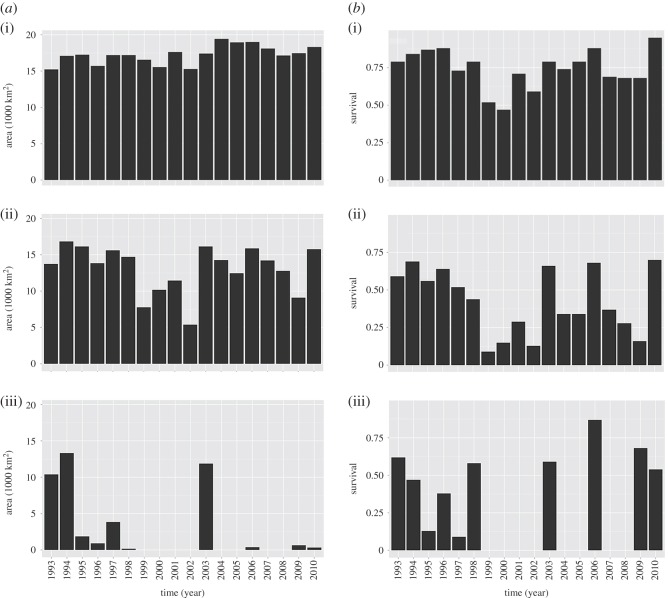
Timeseries of spawning area extensions in km^2^ (*a*) and corresponding Baltic cod oxygen-related egg survival (*b*) for different water density (buoyancy)levels: (i) 1009, (ii) 1011, (iii) 1013 kg m^−3^.

Figure 6 illustrates the timeseries of the suitable spawning habitat extension and the oxygen-related cod egg survival probability in spring from 1993 to 2010 for different egg buoyancy levels. For eggs spawned by old, large females the habitat extent was relatively constant ranging from 15 000 to 20 000 km^2^ over the entire timeseries. In contrast, for eggs of medium-aged females, the available habitat was more variable with maximum values of 18 000 km^2^ during the beginning of the 1990s and mean levels of about 12 000 km^2^ during the late 2000s. Habitat suitability for this female age category strongly decreased between 1999 and 2002. Finally, habitats for eggs of young spawning females (1013 kg m^−3^) were to a larger extent only available after the major Baltic inflows in 1993 and 1994 (10 000–15 000 km^2^), with minor or no suitable habitat availability over the remaining timeseries.

The oxygen-related cod egg survival probability timeseries yielded a higher degree of interannual variability compared with the corresponding habitat suitabilities. Within the suitable spawning area survival probability of eggs produced by the oldest females varied between 0.5 and 1.0, whereas for the eggs of the mid-age and young females, survival was highly variable (0.1–0.7). Highest values occurred during periods following major Baltic inflows (1993, 1994 and 2003). Only these inflow events allowed the youngest cod to successfully contribute to reproduction. During these periods, the magnitude of their egg survival probability is similar to that observed for the mid-aged spawners.

**Figure 7.**
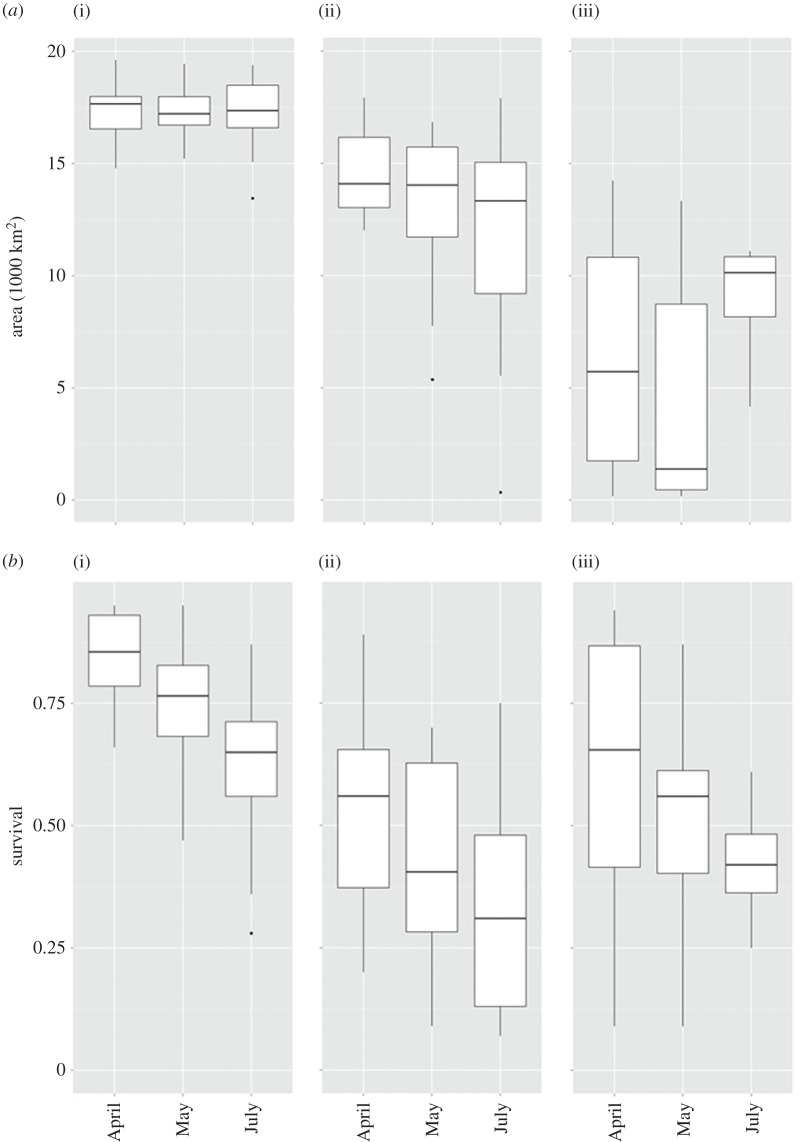
Seasonal differences of spawning area extensions in km^2^ (*a*) and corresponding Baltic cod oxygen-relatedegg survival (*b*) for different water density (buoyancy) levels: (i) 1009, (ii) 1011, (iii) 1013 kg m^−3^.

Seasonal differences in the cod spawning environment (figure 7) indicated a substantial decrease of oxygen-related egg survival probability from April to July in all female age categories. This was due to oxygen depletion during the spawning season. In contrast, such a seasonal decrease in spawning habitat extension was only found for mid-aged and young spawning cod, whereas the habitat for large spawning fish remains almost constant throughout the spawning season. The calculated increase in habitat extension for the young spawning females at the end of the spawning period is insignificant (*n*=4; figure 7) and is mainly owing to inflowing warm and oxygen-rich water masses during summer months in the early 2000s.

**Figure 8.**
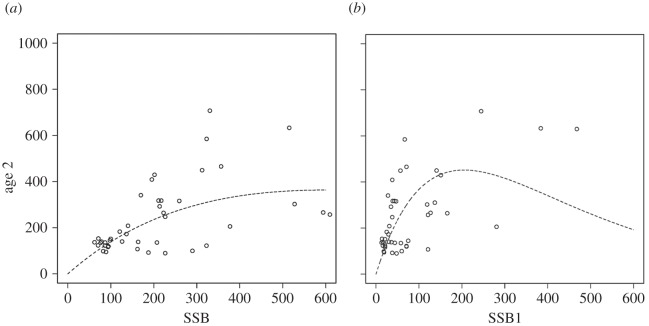
Stock recruitment functions for traditional SSB estimates (*a*) and eSSB, taking into account age-specific egg survival under variable hydrographic conditions (*b*).

The resulting stock-recruitment curve when using eSSB is steeper at low stock sizes and shows a more pronounced density dependence at high stock sizes (figure 8). The Ricker model using eSSB results in a better fit (*r*^2^=0.39) when compared with the standard Ricker model (*r*^2^=0.28) when inspecting the linearized model. All parameters are highly significant.

## 4. Discussion

The novel concept of the eSSB indicator introduced here, and its improved performance relative to traditional SSB as indicator of recruitment, underscored that indices including environmental information can improve biological forecasts and will thus benefit resource management decisions. These benefits may be particularly pronounced in the environmentally challenging conditions of hypoxic area ecosystems (e.g. Baltic and Black Seas), but also mirror the recent broader discussion regarding benefits of ecosystem-based versus single species approaches in fisheries management [36].

The results of our study highlighted the presence of spatio-temporal dynamics in the eastern Baltic cod spawning habitat extension as well as spawning habitat quality in terms of environmentally driven egg survival. These findings underscore the potential role of environmental conditions for the recruitment success of this stock. Similar conclusions were already obtained previously by Hinrichsen *et al.* [37] based on the single station derived reproduction volume concept as a simple indicator describing an overall integrated measure of spatially varying process information [35]. However, importantly, in contrast to this previous approach, we are now able to disentangle the interactions between spawning stock-related reproductive effort and hydrographic parameters in causing variable recruitment. This approach is ‘custom-made’ for the Baltic. It may also be applicable in the Black Sea, which is comparable in physical settings and ecosystem structure. Here, as in the Baltic Sea, the water column is strongly stratified as salinity increases with depth and the deep water is hypoxic/suboxic/anoxic. Fish eggs are semi-/mesopelagically distributed [5] as in the Baltic Sea. In contrast, the present concept is not applicable to management of fish stocks in the large OMZs of the Atlantic and Pacific oceans, because the physical setting and ecosystem structure of these OMZs differ from that of the Baltic Sea. Here, salinity decreases with depth and, hence, semi-mesopelagic spawning behaviour is not evident, and the vertical distribution of eggs is completely different with no steady-state vertical distributions of semi/mesopelagic eggs in these regions [38–41].

By considering the direct link between Baltic cod female age, egg buoyancy and egg survival, our results revealed large changes in the horizontal extent of the suitable female age-dependent spawning habitat and the oxygen-dependent egg survival probability between a period following a Baltic inflow event and a stagnation situation. Stagnation periods in the Baltic have long been recognized as potential threat to cod recruitment [42,43]. Here, we extended these findings by showing that the more buoyant eggs of large females retain a relatively large suitable habitat and survival probability even during a stagnation period, whereas eggs of medium and small females faced strongly reduced habitat and survival probability. The difference of the combined effect of habitat suitability and potential egg survival was about twofold between eggs of large and small females after an inflow event, but much larger during stagnation (approx. 35-fold).

Including these considerations in the novel indicator eSSB improved the explained variability in recruitment to 39%, relative to 28% explained by SSB, i.e. performance of ‘traditional’ SSB was improved by more than one-third. This stressed the biological relevance of the added parameters and the potential usefulness for fisheries management. At the same time, even after including the spawner-age effect, remaining variability (61% relative to 72% unexplained by traditional SSB) in stock recruitment was still large.

This was probably owing to a number of other processes which have been implicated in cod recruitment success, including varying egg predation by sprat and herring [44,45], maturity [46] and fertilization success [47,48]. Moreover, during the larval stage, survival may be critically affected by reduced suitable prey abundance [49] and by variable larval drift [50,51]. Furthermore, as obtained from [23], the inverse relationship between egg diameter and neutral buoyancy levels lacks a considerable amount of explained variability (*r*^2^=0.49). This implies that other factors potentially contribute to variations in egg buoyancy. Improved egg buoyancy values could be obtained if the ratio of chorion volume/egg volume of individual eggs is explicitly known [38,39], i.e. the water content in the yolk (hydrolyzation of the proteins) could vary and influence egg buoyancy. However, compared with the experimentally derived relationship between egg diameter and buoyancy, the field-based relationship revealed a much higher level of explained variability. Furthermore, individual females produce eggs of different diameters (i.e. buoyancies) within the spawning batch of cod [23,24], with in general a higher survival potential of the early batches compared with the later ones. Incorporating an average decrease in egg size with batch number and the expected decrease in buoyancy would lower egg survival estimates over the spawning season even further, but the magnitude of this effect is difficult to quantify. For this reason, we calculated simplified egg buoyancy levels (averages over all spawning batches) for each female age class based on experimental studies [23]. A comprehensive field-based validation is presently still missing, as detailed information on the abovementioned factors is lacking and only limited datasets have been analysed so far [52,53].

Our results directly indicate the importance of age-class specific reproductive effort in relation to environmentally based egg survival. Because of strong inter- and intra-annual variations of the central Baltic environment, the eastern Baltic cod stock is a perfect example for a direct link between female size (age), egg survival potential and recruitment. Any processes that further change the size/age structure of a stock towards smaller size, such as fisheries induced evolution towards, for example, earlier maturity [54,55], would exacerbate the reduction of eSSB relative to the total population.

Furthermore, the results emphasize the importance of an age-structured and sex-specific abundance index for the Baltic cod stock, similar to ideas suggested for the evaluation of Good Environmental Status within the European Marine Strategy Framework Directive [56–58]. Under the current exploitation regime of eastern Baltic cod, the fish population structure is highly biased towards smaller size and age classes, i.e. ‘big fat female fish’ are rare, but might form (if present) an ecological insurance against increasing hypoxic area extensions. This supports the idea of the ‘importance of leaving the big ones’ [59], and call for the additional inclusion of, for example, a ‘large fish indicator’ [60] in the current stock assessment and management.

Specifically, we propose to use the percentage of old, large females in the stock as a measure of stock resilience to unfavourable environmental conditions. Our results also indicate an effect of stock structure on economic performance of the stock. The same stock biomass comprising smaller individuals will produce less and more variable recruitment when compared with a stock made up of larger fish. This unfavourable age structure has to be paid for in terms of lower sustainable harvest, and a risk premium, quantifying the costs of increased cod biomass variability [61,62].

The key assumptions underlying this paper are that large, older females, on average, produce larger, more buoyant eggs compared with smaller less buoyant eggs spawned by smaller, younger females [23], and that there is a strong negative correlation of egg diameter with neutral egg buoyancy. In addition, there are reasonable indications that the feeding conditions for adult eastern Baltic cod might have changed during the most recent decade [63,64]. Future work repeating laboratory experiments to assess potential changes in female age-dependent egg production and in the egg size relationship and to validate our findings under the present feeding regime would be useful. Other planned future activities include the analysis of female age-dependent egg size/buoyancy relationships in combination with hydrographic data and spatial cod egg distributions to provide information on the availability, accessibility and vulnerability of the different adult female cod age (size) categories in relation to fishing activities during spawning periods. For longer time periods, a lack of data exists to entirely resolve the environmental conditions for the major spawning season. To overcome this problem, future applications of our method will benefit from a new hydrodynamic model version for the Baltic Sea, which will be able to successfully simulate both oxygen consumption and oxygen replenishment of pelagic as well as benthic zones [65].

Spatial planning based on high-quality habitat information of the key species in focus is required [66]. Here, the temporal dynamics of the ecosystem components influencing the life cycle of key species must be captured in the assessment of habitat suitability and extension and must be taken into consideration when applied in relation to management. Furthermore, long- to medium-term fluctuations of habitats caused, for example, by climate change need a regularly updated assessment of habitat characteristics to ensure that they reflect the ecosystem under management. Baltic cod egg buoyancy level structured spawning habitat extension and quality could be quantitatively considered as an *essential fish habitat* defined as *those waters and substrates necessary to fish for spawning, breeding, feeding or growth to maturity* according to the US Magnuson–Stevens Act [67].

Because hypoxic area extension in the Baltic Sea is expected to increase, expected habitat losses (e.g. size of spawning grounds) of ecologically and economically important fish species should in future be considered for management decisions regulating fishing pressure as well as harvest rates. Results obtained from IPCC future modelling scenarios show increasing oxygen concentrations in the large OMZs of the Atlantic and Pacific oceans, but predicted a decrease in higher latitudes including the Baltic Sea [8], as the predicted increase in temperature leads to a general decrease in the oxygen solubility of seawater [52,68].

Advanced and effective ecological indicators (e.g. basin-wide integrated egg survival probability instead of the single station-based reproduction volume of Baltic cod) should have, in focus, the requirements of EU Marine Strategy Framework Directive indicators, and will contribute to commercial fish stocks within safe biological limits as well as exhibiting a population age and size distribution that is indicative of a healthy stock. As fishery stock assessment and projection tools have improved within the last few years, it has become easier to incorporate environmental and ecological data into population dynamic models [69] and stock management frameworks.

## Supplementary Material

appendices_revised.docx the file contains description of different methods: a) objective analysis - horizontal mapping b) oxygen-related cod egg survival function c) age-specific weighting for effective spawning stock biomass calculations

## Data accessibility

To make the results of our article fully reproducible, all supporting data are accessible in the Dryad repository: http://dx.doi.org/10.5061/dryad.2pm6f.
