# Supplementary material for: Oxygen depletion in coastal seas and the effective spawning stock biomass of an exploited fish species
Source: R Soc Open Sci. 2016 Jan 13;3(1):150338. doi: 10.1098/rsos.150338 (PMC4736919; doi:10.1098/rsos.150338)
Supplement: appendices_revised.docx the file contains description of different methods: a) objective analysis - horizontal mapping b) oxygen-related cod egg survival function c) age-specific weighting for effective spawning stock biomass calculations [file rsos150338supp1.docx]

**Appendix 1:**

The horizontal interpolation technique uses a spatial isotropic Gaussian covariance function of measurements:

$f\left( r \right)=\sigma^{2}\exp({-r^{2}}/{R^{2})}$ (1)

with *R* being the autocorrelation scale parameter and *σ* the variance and *r* the distance between data points. *R* was determined from the fit of this covariance function to the raw covariances of the field observations.

**Appendix 2:**

The results are based on a modeled egg survival function obtained from experimental data [28], not on direct field observations:

$\text{y}_{o}=\text{ 1}00\left(1-\left(\left(\text{1 }-\text{ e}-\left(0.\text{71x}\right)\right)11.63\right)\right),$ (2)

where y_o_ is the oxygen-dependent survival probability before hatch and x is the oxygen content in ml l^–1^. Ambient oxygen contents were obtained from our hydrographic database. The function (r² = 0.94) describes a sigmoid curve with almost total mortality at 2 ml l^–1^ oxygen content, ~50% mortality at 4 ml l^–1^, and <10% mortality at 7 ml l^–1^. Generally, oxygen-dependent survival at egg buoyancy levels was calculated with respect to the above mentioned threshold levels in temperature, salinity and oxygen concentration.

**Appendix 3:**

Age-specific weighting factors for eSSB calculation were applied as follows:

$\begin{matrix} \text{SSB }=\text{ }\sum_{s=1}^{8} \gamma_{s} w_{s} x_{st} \\ \text{eSSB }=\text{ }\sum_{s=1}^{8} \sigma_{s} \gamma_{s} w_{s} x_{st} \end{matrix}$ (3)

where *x_st_* are the stock numbers at age *s* in year *t*, *w_s_* is weight at age, $\gamma_{s}$maturity at age and $\sigma_{s}$ is the age-specific survival factor (age-specific recruitment weighting factor, see Table 2). Data on stock numbers, weight in the stock, and maturity were taken from [8]. In order to assess the usefulness and validity of this new measure we then calculated the amount of variability in recruitment explained by “normal” SSB and by the eSSB. to assess the usefulness and validity of this new measure.

We tested whether the use of the newly-compiled eSSB time-series would improve the fit of a traditional Ricker-type stock-recruitment model, using data from the most recent accepted stock assessment in 2013 [8].

Nonetheless, we first tested which stock-recruitment function yielded the best fit for the traditional SSB estimates. The Ricker-type model fitted the data best, however, being only slightly better than the Beverton-Holt or the smoothed hockey stick model. Therefore, we concentrated on the Ricker model and tested whether the use of the newly-compiled eSSB time-series would improve the fit:

(3a)

vs.

(3b)
